# RECIPROCITY AND INDIVIDUAL DIFFERENCES IN DIET-RELATED SPOUSAL INVOLVEMENT AMONG OLDER COUPLES MANAGING DIABETES

**DOI:** 10.1093/geroni/igz038.977

**Published:** 2019-11-08

**Authors:** Kristin J August, Caitlin S Kelly, Charlotte H Markey, Shibin Yan

**Affiliations:** 1 Rutgers University, Camden, New Jersey, United States; 2 University of Utah, Salt Lake City, Utah, United States

## Abstract

Spouses are commonly involved in supporting and regulating their partners’ diabetic diet. Older spouses also may be managing their own condition that requires changes to their diet. Little is known about the extent to which this spousal involvement is reciprocal and if there are individual differences in the provision of diet-related support and control (regulation). This study sought to understand the reciprocity of diet-related support and both positive and negative forms of control, whether personality traits predicted the provision of diet-related spousal involvement, and whether gender moderated these associations. We used data from a cross-sectional survey of 150 couples (50+ years) in which one member had type 2 diabetes (Mage=65.94; Mmarital length=33.46; 50.7% male patients; 58.7% of spouses had a condition that required changes to their diet). The provision and receipt of social support and two forms of social control were moderately correlated within individuals (patient rs=.36-.45, ps<.001; spouse rs=.41-.73, ps<.001). Using Actor Partner Interdependence Models that controlled for age and responsibility for managing meals, results revealed that being more extraverted or having a partner who was more extraverted was associated with greater involvement, whereas being more neurotic or having a partner who was more neurotic or had more interpersonal problems was associated with less positive and greater negative involvement. Associations were particularly pronounced for men. Results suggest that there is moderate amount of reciprocity, as well as personality differences, in diet-related spousal involvement. These findings highlight the importance of targeting both members of the couple in diabetes intervention efforts.

